# Persistent homology-based segmentation tool for membrane images

**DOI:** 10.1016/j.crmeth.2026.101366

**Published:** 2026-03-26

**Authors:** Haruhisa Oda, Yusuke Imoto

**Affiliations:** 1 Department of Preventive Medicine, Graduate School of Medicine, The University of Tokyo, Tokyo, Japan; 2 Institute for the Advanced Study of Human Biology, Kyoto University, Kyoto, Japan

**Keywords:** persistent homology, topological data analysis, embryo membrane image, segmentation, tracking

## Abstract

While advances in machine learning have enabled automated cell segmentation, users often face challenges in parameter tuning until reaching their desired results. To address this issue, we developed PomSeg, a membrane segmentation method based on persistent homology. Since persistent homology captures topological features of input data, PomSeg parameters reflect cell shape information, enabling intuitive and efficient parameter tuning. This adaptivity, together with stability for noise of persistent homology, enables robust application of PomSeg to various image types, including coarse-resolution data. By applying PomSeg to early mouse embryo membrane images and other publicly available datasets, we demonstrated its flexibility, versatility, and robustness, along with agreement with ground truth. Additionally, we showed the potential of PomSeg extension by incorporating a machine learning tool in its process. These features make PomSeg a valuable tool for researchers pursuing control and interpretability in segmentation, as well as indicating wider applications beyond a machine learning alternative.

## Highlights

• PomSeg, a persistent homology-based membrane image segmentation tool
• User-centered flexibility and versatility ensures control in image analysis tasks
• High consistency with ground truth and robustness to noise and resolution
• PomSeg offers precision and control for image analysis users

## Motivation

The trade-off between flexibility and automation is a crucial topic in cell segmentation. Machine learning-based tools serve as examples of automation technologies, designed to learn from and adapt to input datasets. However, there is also a significant need for user-centered, flexible tools that have parameters with clear meanings. Persistent homology is an advanced method used to capture topological information from data, and it has been employed to create user-centered cell nuclei segmentation tools. Despite this, there has yet to be a development of persistent homology-based membrane image segmentation tools with user-centered features, even though this image modality is vital for biological studies. Here, we introduce PomSeg, a persistent homology-based membrane image segmentation tool that utilizes both 2D and 3D persistent homology processes.

## Graphical abstract

**Figure:**
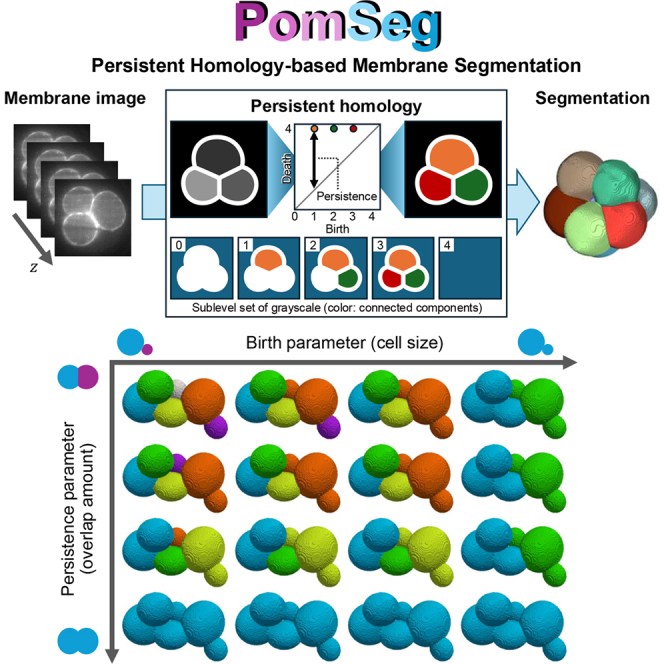

## Teaser

Oda and Imoto develop PomSeg, a persistent homology-based membrane image segmentation tool. PomSeg enables user-centered flexible parameter tuning and is applicable to wide range of membrane images with different levels of noise and resolution. With these beneficial properties, PomSeg provides advantages for users seeking precision and control in image analysis.

## Introduction

Cell segmentation and tracking are essential tools in biological image analysis^1^ that enable researchers to quantitatively analyze dynamic cellular properties, including shapes, positions, connectivity, volumes, and movements. These tools provide fundamental insights into biological processes such as development and disease.^2^^,^^3^ Recent advances in image acquisition technologies have increased the demand for quantitative analysis. Therefore, more researchers are now aware of the benefits of cell segmentation and tracking tools.

Several approaches to cell segmentation have been developed. A traditional example is manual segmentation, which biologists often perform using platforms like Fiji.^4^ Additionally, biologists can utilize modules in CellProfiler^5^ and similar tools. Some methods come with integrated image acquisition methods, as seen in the work of Fernandez et al.^6^ Recent developments in machine learning, particularly deep learning, have transformed the field into a widely utilized tool among biologists, as demonstrated in previous studies.^7^^,^^8^^,^^9^^,^^10^^,^^11^

A significant issue in cell segmentation tools is the trade-off between flexibility and automation.^10^ Machine learning-based tools are usually data-centered. They are designed to learn from and fit to the input dataset, thereby automating the analysis flow. On the other hand, there is also a need for flexible tools. Here, what we mean by flexible tools are user-centered tools that have parameters with clear meanings. This allows users to interact with and understand these parameters.

As a user-centered tool, we focus on topological data analysis (TDA). TDA is a data analysis tool to describe topological information of data based on geometry or topology theory. Persistent homology^12^^,^^13^ is a cutting-edge method in TDA, developed in the 21st century, recognized for its versatility and practicality. While persistent homology was initially applied to non-biological fields, like material science,^14^ its applications in biomedical science are gaining attention, particularly for its ability to extract structural patterns in living systems. For example, persistent homology has been utilized in the analysis of hepatic lesions,^15^ brain arteries,^16^ *C. elegans* movements,^17^ and transcriptomics.^18^ Notably, its application to image segmentation^19^^,^^20^^,^^21^^,^^22^^,^^23^ has shown great promise, with certain studies^21^^,^^22^^,^^23^ focusing on user-centered tools that feature parameters with clear geometric meanings.

However, previous persistent homology-based segmentation tools are largely limited to cell nuclei, especially those that are easily binarized. Though this imaging modality is important, it is true that these images are largely similar and represent only a portion of the broader range of life science images. In addition to nucleus images, another major imaging modality involves membrane images, particularly the 3D images of embryo cell membranes in developmental biology. The importance of this image modality is supported by the existence of multiple works dealing with embryo cell membrane segmentation^24^ for different species, such as nematodes,^25^^,^^26^ ascidian,^27^^,^^28^ fly,^29^^,^^30^ zebrafish,^31^^,^^32^ and human,^33^^,^^34^ for multiple species,^11^^,^^35^^,^^36^ and for different experimental settings.^37^ Therefore, developing a framework for applying persistent homology to cell membrane images could greatly expand the versatility of the persistent homology-based cell segmentation.

In this paper, we present PomSeg (a persistent homology-based membrane segmentation) that can process 2D and 3D membrane image segmentation. We will address the challenges associated with typical membrane images, such as varying signal intensities and overlapping structures, by combining different types of persistent homology processes (2D grayscale and 3D distance-based filtrations) with image analysis filters and transformations. Our primary application target is membrane images of mouse pre-implantation embryos,^35^ along with additional analyses on peri-implantation embryos^38^ and various open-source images. We will discuss the benefits of PomSeg based on flexibility, versatility, robustness, and consistency with ground truth. Flexibility refers to its user-centered design, featuring interpretable parameters. Versatility will be demonstrated by its capability to accept inputs with various image conditions, such as intensity, size, and shape. We will assess its robustness in relation to different time points and *z*-resolutions. Consistency with ground truth will be evaluated by comparing segmentation volumes in time series data. In addition to these four features, we will finally provide examples of its extensibility by modifying certain components of PomSeg to meet specific needs, which lays a foundation for future research aiming for broader significance. PomSeg source code and GUI standalone application are made publicly open at https://github.com/TopologicalBird/PomSeg.

## Results

### PomSeg identifies cells based on topological structures of cell images

**Figure 1:**
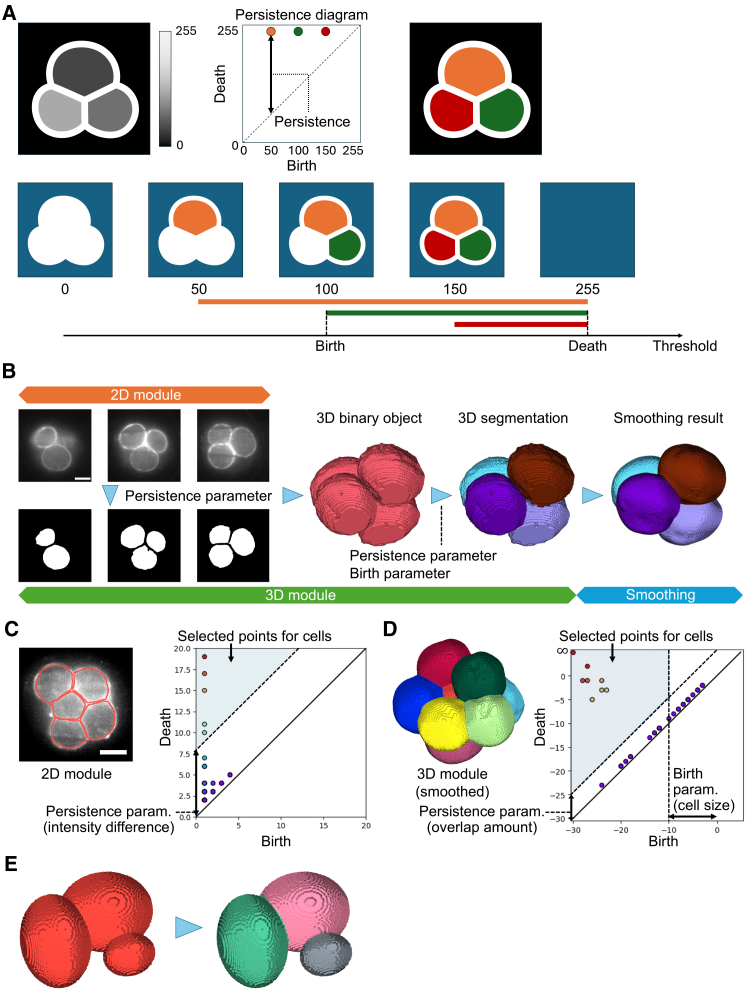
Overview of persistent homology and PomSeg framework (A) A visual explanation of the persistent homology process; a target grayscale image (top left), its persistence diagram (top middle), detected region labels (top right), and tracking of sublevel set images (bottom). The values on the bottom figures are the thresholds of sublevel set images. Each color corresponds to a point in the persistence diagram, and the colors are common among all figures. (B) An illustration of PomSeg’s analysis pipeline. The 2D module is illustrated in the left part, with the upper row showing the original images and the lower row showing the binary masks generated by the 2D module. The 3D module is a combination of the left and right parts. The 3D binary object is the result obtained from the 2D module, and the second right most segmentation is the result of the 3D module. We can apply postprocessing to get smoothed cell segmentation as in the right most image. Sample image: 4-cell stage mouse embryo from Fabrèges et al.^35^ (35th time point). The resolution of the *xy*-plane is 0.416 μm/px, and the resolution of the *z* axis is 1.338 μm/px. Scale bars, 25 μm. (C and D) Example images showing PomSeg’s parameter tuning. (C) and (D) show 2D module and 3D module (after smoothing), respectively. The points inside the blue regions in the persistence diagrams are selected and visualized using inverse analysis on the original image. Sample images: 8-cell stage mouse embryos from Fabrèges et al.^35^ (58th and 53rd time points). The resolution of the *xy*-plane is 0.416 μm/px, and the resolution of the *z* axis is 1.338 μm/px. Scale bars, 25 μm. Red contours in (C) were drawn by an OpenCV library in Python. (E) An application of PomSeg to a model cell image with large contact surfaces. PomSeg can effectively separate cells with ambiguous boundaries. All the births and deaths in the figures mean the births and deaths of the points in the persistence diagrams.

Persistent homology quantitatively extracts topological information, such as connected components, holes, and cavities, from data. For instance, when analyzing a grayscale image, where the value on each pixel is one-dimensional, we focus on the sublevel sets of the image, namely the pixels with intensity values less than a threshold value. The (0th) persistent homology algorithm then tracks the birth, death, and persistence (death minus birth) of connected components as the threshold value changes. The output of this process, called a persistence diagram, is a two-dimensional scatterplot that displays the pairs of threshold values indicating the births and deaths of these connected components (Figure 1A). Another visualization of the output is called a barcode, where we visualize the birth and death of each point in the persistence diagram with an interval (Figure 1A, bottom). Persistent homology is characterized by several important features: the parameters and outputs are geometrically interpretable (“white box”), the output reflects the topological sizes of the features, and it is robust to measurement noise (stability theorem^39^). Additionally, the topological information in the output can be mapped back to the original data through a process known as inverse analysis.^40^ Persistent homology can also be extended to any dimension, and these characteristics have been formulated mathematically. With these properties, persistent homology is a strong candidate for building an effective membrane segmentation tool.

PomSeg, a cell membrane image segmentation tool, employs persistent homology in a hierarchical manner. When provided with 2D image data, PomSeg utilizes grayscale intensity-based persistent homology to produce cell segmentation results (Figure 1B; see “PomSeg 2D module: 2D persistent homology” in STAR Methods for mathematical details). If a set of 2D slice images obtained from 3D cell imaging is the input, PomSeg can generate, from a set of 2D segmentation results, a 3D segmentation result (Figure 1B) using a 3D distance-based persistent homology calculation. Finally, with a smoothing process, we can get smooth cell segmentation results as in the right most image in Figure 1B. PomSeg retains the beneficial properties of persistent homology: its parameters are interpretable, and the pixel intensity difference in 2D or cell size and overlap in 3D are reflected by the birth and persistence in the persistence diagram. Therefore, users can easily adjust parameters according to their target image data, as the parameters can be set based on the intensity difference for 2D and cell size and cell overlap amount intended for detection for 3D (Figures 1C and 1D). Additionally, it is robust to noise, and the algorithms for both 2D and 3D segmentation are seamlessly integrated. Moreover, PomSeg utilizes a transformation that identifies object centers from image topology (distance transform, “PomSeg 3D module: Distance transform” in STAR Methods), allowing it to effectively separate cells with ambiguous boundaries, such as cells with large contact surfaces (Figure 1E). As a result, using PomSeg for cell image data enables users to obtain accurate cell segmentation results with greater flexibility.

### 2D module versatility tested with different biological systems and image conditions

**Figure 2:**
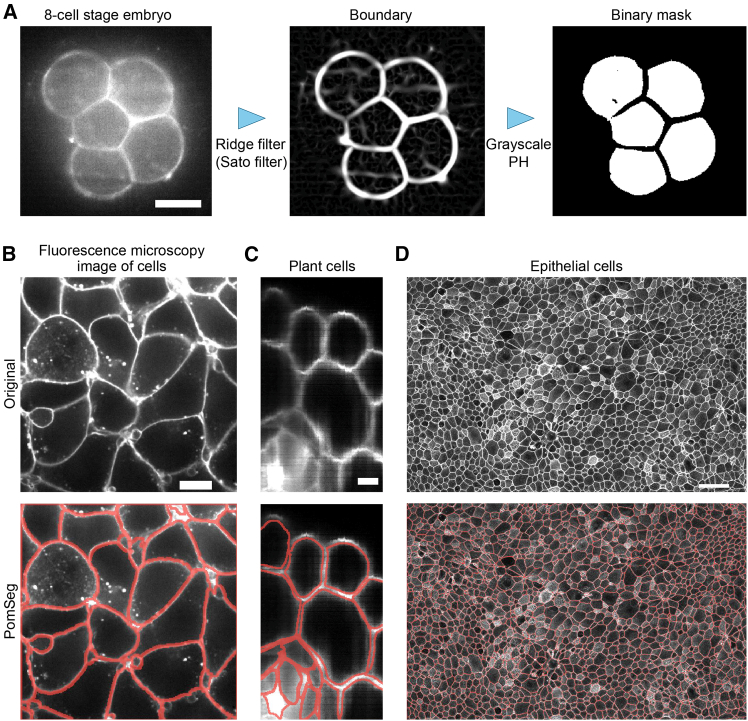
2D module versatility tested with different biological systems and image conditions Examples showing the effectiveness of PomSeg 2D module in various biological systems and image conditions: (A) an 8-cell stage mouse embryo from Fabrèges et al.^35^; (B) a fluorescence microscopy image of cells in scikit-image; (C) a plant cell image (lateral root primordia of *Arabidopsis thaliana* in https://osf.io/6x4gz); and (D) a human bronchial epithelial cell image in https://github.com/tkphung/CellSegmentation/tree/main. In (A), the left image is the original image, the middle is the one after preprocessing, and the right is the binary mask generated by the 2D module. In (B), (C), and (D), the upper images are the original images, and the lower images are the visualizations of membranes detected by the 2D module (red lines). Image resolutions are (A) 0.416 μm/px for *xy*-plane, (B) 0.26 μm/px for *xy*-plane, (C) 0.1625 μm/px for *xy*-plane, and (D) 0.293 μm/px for *xy*-plane. Scale bars: (A) 25 μm, (B) 10 μm, (C) 5 μm, and (D) 50 μm. Red contours were drawn by an OpenCV library in Python.

In this section, we examine the effectiveness of PomSeg 2D module across various biological systems. Membrane contours were delineated by applying an OpenCV library in Python to the binary mask created by the PomSeg 2D module. Also, when generating images with high intensity values on the cell boundaries as in the center of Figure 2A, we used a ridge filter (Sato filter,^41^ See “PomSeg 2D module: preprocessing” in STAR Methods).

We demonstrate the applications of the 2D module to different 2D biological images: an 8-cell stage embryo (Figure 2A), a fluorescence microscopy image of cells in scikit-image (Figure 2B), a plant cell image (Figure 2C), and an epithelial cell image (Figure 2D). These examples not only highlight the biological diversity of the application targets but also demonstrate the module’s versatility in accommodating different imaging conditions. For instance, Figure 2B features cells with various shapes, Figure 2C displays a wide range of intensities, and Figure 2D includes numerous cells with different shapes and intensities. In Figures 2A–2C, where original images are relatively clear, PomSeg gave a good membrane detection or segmentation. While the results in Figure 2D included small errors, showing some internal noise lines (see Figure S1), PomSeg effectively detected the membrane regions without changing the overall analysis flow.

A key parameter in this 2D module is the persistence parameter (Figure 1C), where persistent homology is calculated using the grayscale intensity values in the membrane images. Therefore, this parameter determines the minimum intensity difference between a structure’s interior and its contour that is required for the structure to be detected as a cell. Consequently, this parameter possesses an understandable meaning, which ensures user-centeredness.

### 3D module robustness tested with time series data and downsampled images

In this section, we present an analysis of time series data derived from 8-cell stage mouse embryo images used in Fabrèges et al.^35^ This time series has 34 time points taken every 15 min during the 8-cell stage. Each time point has a 3D image of size 254∗254∗88. The resolution of the *xy*-plane is 0.416 μm/px, and the resolution of the *z* axis is 1.338 μm/px.

**Figure 3:**
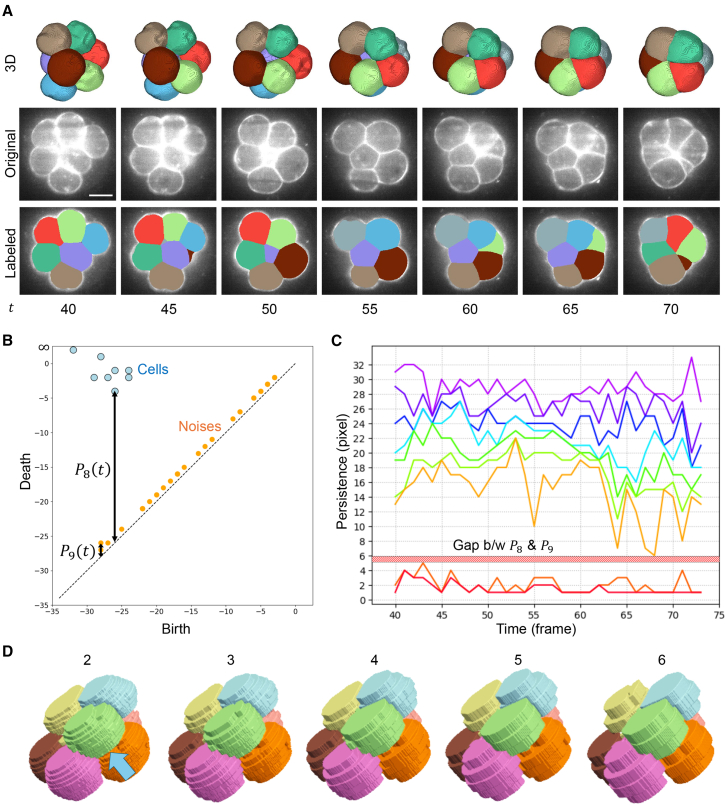
3D module robustness tested with time series data and downsampled images (A) The segmentation outcomes resulting from our analytical workflow. The top row shows the resulting 3D labels (smoothed), the second row shows the 2D slices of original images, the third row shows the smoothed labels overlayed on the original images, and the bottom row shows time. Full video is available in the PomSeg repository. Scale bars, 25 μm. Same color labels represent identical cells. (B) An example of the resulting persistence diagrams generated by the 3D module at the 53rd time frame. *P*_*i*_(*t*) shows the *i*th largest persistence at *t*th time frame (*t* = 53). The blue and orange points are the points corresponding to the 8 cells to be detected and the other background artifacts (noises), respectively.(C) The time variations of the 2nd to 10th largest persistence. The gap between *P*_8_ and *P*_9_ indicates the existence of a single persistence threshold that robustly separates cells from background structures for all the time points in this time series data. One time frame corresponds to 15 min. (D) PomSeg segmentation results with lower *z*-resolutions. We tried downsampling intervals of 2–6. PomSeg was able to separate touching cells in lower *z*-resolutions. Same color labels represent identical cells. All the births and deaths in the figures mean the births and deaths of the points in the persistence diagrams.

First, we used all 34 time points. We fixed the parameters of the PomSeg algorithm, except for the persistence parameter employed in the 3D module, which we will mainly examine here. Although the user-centeredness that allows intuitive parameter tuning is the main advantage of PomSeg, this constraint was applied to facilitate benchmarking. The 3D module of PomSeg successfully separated 8 cells from time-series membrane images using a single, fixed parameter setting (Figure 3A; see a video in the PomSeg repository). This was possible because the 8 most persistent points in the persistence diagram, presumed to correspond to the 8 actual cells, had significantly longer persistence than all others (Figure 3B; the 53rd time frame). This clear separation of persistence values was consistently observed across almost all time points (Figures 3C and S2). We manually verified that the points in the persistence diagrams with the 8 longest-persistence corresponded to the actual cells, rather than background artifacts, by performing inverse analysis of persistent homology (“PomSeg 3D module: 3D persistent homology and finalizing segmentation” in STAR Methods) across all time points. Moreover, we observe a gap between the lower bound of the 8th persistence (6) and the upper bound of the 9th persistence (5) across time (Figure 3C). These results demonstrate not only the existence of a single persistence threshold that robustly separates cells from background structures, but also the noise robustness of PomSeg, as it treats each time point as an independent dataset potentially containing different noise. These results underscore the efficacy of PomSeg in the analysis of this time series dataset. Even in instances where these optimal results do not hold, PomSeg can still be effectively employed through appropriate parameter adjustment, which will be explored later when we apply PomSeg to different cell number stages.

Next, we investigated the robustness of PomSeg under variations in input data resolution. To simulate lower *z*-resolution, we generated downsampled images from the 53rd time frame by sampling *z*-slices at fixed intervals. We tested multiple intervals, specifically integers between 2 and 6, for the downsampling process. Given an integer *i* representing the interval and the original set of *z*-slices 0, 1, 2, 3, 4, 5, 6 …, we can create a lower-resolution stack by selecting slices such as 0, *i*, 2*i*, and so on. Additionally, we can alter the starting slice to obtain different lower-resolution stacks, such as 1, *i*+1, 2*i*+1, etc. In the original 53rd time frame slices, there was a 2-slice gap between the two vertically aligned cells. Excluding this gap when creating lower-resolution stacks makes the segmentation task more challenging, as the two cells touch each other. To evaluate PomSeg under these difficult conditions, we generated the downsampled images such that the slice samplings consistently skipped the gap by shifting the starting slice. For the interval of 2, we could not exclude the gap by merely shifting the starting slice (due to the original 2-slice gap), so we manually removed the slice containing the gap. This downsampling resulted in two cells touching each other for all the downsampled images, as highlighted by the arrow in Figure 3D. PomSeg successfully segmented cells for all the intervals (Figure 3D). This demonstrates the robustness of PomSeg against images with lower *z*-resolution.

### Consistency of PomSeg result volumes with ground truth volumes

**Figure 4:**
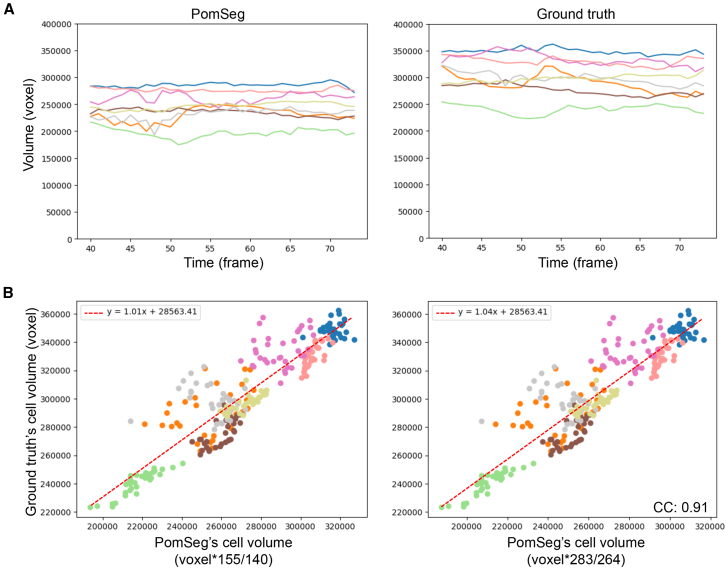
Consistency of PomSeg result volumes with ground truth volumes (A) A comparison of two tracking results of PomSeg (left) with the ground truth result (right) from the previous study.^35^ Colors common to both figures indicate the volume change of the same cell. (B) The scatterplots of the cell volumes evaluated by PomSeg and the ground truth at all the time frames. The colors in the scatterplots correspond to the colors in the tracking results (A). To reduce the effect of *z* axis interpolation method differences, we modified the raw voxel count with the range of *z* values where cells exist (left) and the *z* axis range of the images (right). The correlation coefficient (CC) for PomSeg and ground truth is 0.91.

We tracked segmented cells along time points using a minimum weight maximum matching algorithm, which is a graph matching algorithm to match two groups with the minimum total weight on the edges used for the matching. Here, we refer to the analysis results from Fabrèges et al.^35^ as our ground truth. These segmentation results had been generated using PlantSeg and further refined with manual modifications to ensure consistency with actual cells. We evaluated PomSeg’s tracking results by comparing its cell volume histories with the ground truth (Figure 4A). One possible cause of the voxel number difference between PomSeg and ground truth is that the methods for *z* axis interpolation or scaling are different, and the ground truth result has more *z* slices (283) compared to PomSeg (264). Also, the ranges of *z* values where labels exist in the first time frame are 155 for the ground truth and 140 for PomSeg. When comparing the volumes measured by PomSeg and ground truth, we used these values to reduce the difference caused by interpolation methods, which are unrelated to segmentation quality (Figure 4B).

We can find a strong consistency between the two methods. We manually colored the tracking cell volumes corresponding to the same cells (Figure 4A) and investigated their correspondence. The correlation coefficients between PomSeg and the ground truth for all the cell volumes across time points was 0.91 (Figure 4B), and the average coefficients of variation across time points were 0.033 for PomSeg and 0.032 for the ground truth. These results indicate that PomSeg achieved time-series cell segmentation with a volume distribution correlated with that of ground truth. Further investigation of cell volumes revealed that, in both tracking results, there was a consistently smaller cell at almost all time points. This cell was in the center part of the embryo, which may have a biological significance that could be explored in future works.

### Comparing PomSeg with existing methods

**Figure 5:**
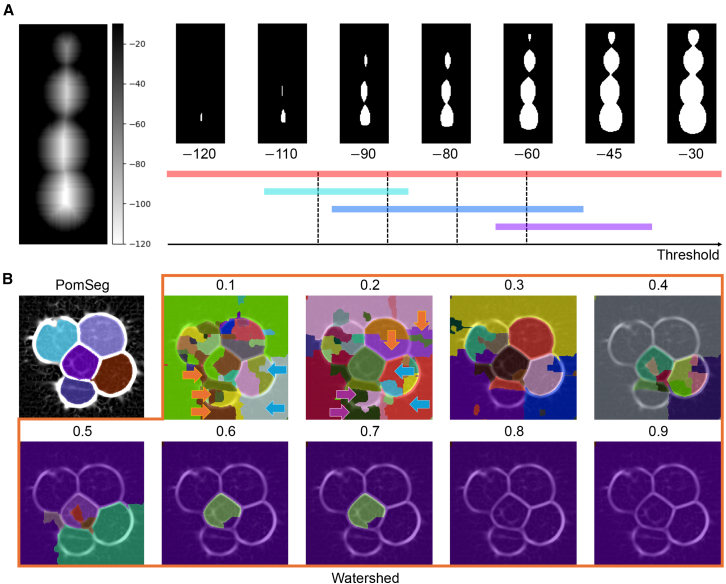
Comparing PomSeg with existing methods (A) An example of four figures that can be detected by persistent homology but cannot be detected by threshold-based approaches. Modified from Figure 4 in Oda et al.^22^ We can set appropriate birth and persistence parameters to detect the four bars corresponding to the four figures, but there exists no single threshold value (dashed lines) that can detect the four figures simultaneously. Also, all the threshold-based information is stored as sections of the barcode. This shows the superiority of the persistent homology-based figure detection when compared to threshold-based approaches. Note that white color shows negative values in this figure. (B) Comparison of PomSeg and a watershed method. PomSeg was able to segment Sato filter-processed images. On the other hand, when applying a watershed tool to Sato filter-processed images, we could not get satisfactory results. Even when several cells were oversegmented, there were still some undersegmented parts highlighted by arrows. This means that PomSeg was able to handle an input that was hard to process with a watershed method.

This section compares PomSeg with existing methods from two perspectives. First, we start from a theoretical perspective. We point out the superiority of persistent homology-based figure detection when compared with threshold-based tools such as binarization, dilation, and erosion.^22^ These threshold-based tools are frequently incorporated in image segmentation tasks (e.g., Wang et al.^42^). We use Figure 5A as our example. The grayscale image in the left part of Figure 5A was generated by the distance transform process from a binary image of four overlapping figures, as we did in the PomSeg 3D module. The barcode in Figure 5A shows the persistent homology calculation result. If we set the absolute value of the birth parameter smaller than the birth of the 4th purple bar and the persistence parameter smaller than the persistence of the 2nd light blue bar, we can detect the 4 figures. On the other hand, threshold-based approaches, in this case, erosions, fail because there exists no single threshold value that can detect the 4 figures simultaneously. This can be seen from the fact that no vertical line can pass through the 4 bars simultaneously (see the dashed lines in Figure 5A). This example shows that, when using threshold-based tools, not only is it difficult to find an appropriate threshold, but there may be cases where no appropriate threshold exists at all. Persistent homology solves this problem while keeping all the threshold-based information.

Next, we have a more concrete perspective by comparing PomSeg with watershed methods, which are often used in segmentation tasks arising in biology.^43^^,^^44^ We used the dataset of membrane images from the 53rd time frame, previously introduced in the 3D module section. For this dataset, we recall that PomSeg achieved accurate segmentation by appropriately setting its parameters (Figure 5B, upper left). We applied a watershed method to the Sato filter-processed image used as an input for the PomSeg 2D module. We tried multiple parameter settings but did not get satisfactory results. Even if some cells were oversegmented, there were other undersegmented parts, resulting in a mixture of over- and undersegmentation (Figure 5B). This means that PomSeg was able to handle an input that was difficult to process with a watershed method.

### Instructions on the parameter tuning of PomSeg using different cell numbers

In this section, we assist users with parameter tuning in PomSeg using different stages of cell numbers in 3D. Since we have already discussed the persistence parameter tuning for the 2D module in previous sections, we now focus on tuning the parameters for the 3D module. For the 3D module, essential parameters for point selection in the persistence diagram are the birth and persistence parameters (Figure 1D). The birth of a point in the persistence diagram corresponds to the size of the object, while persistence reflects the degree of overlap with other objects. In addition to these essential parameters for 2D and 3D persistent homology, we allow users to tune additional parameters related to noise and cell volumes. The explanations of them can be found in Figure S3 and STAR Methods.

We used two additional datasets: 4-cell stage to 11-cell stage time series data, including before and after time points in the previous section, and another time series data, also from the 4-cell stage to the 11-cell stage. We adjusted parameters corresponding to the shape and size of cells in each stage (Tables S1 and S2). Note that we made the parameter change following the status of cells because the goal of this section is not to investigate the robustness of PomSeg.

**Figure 6:**
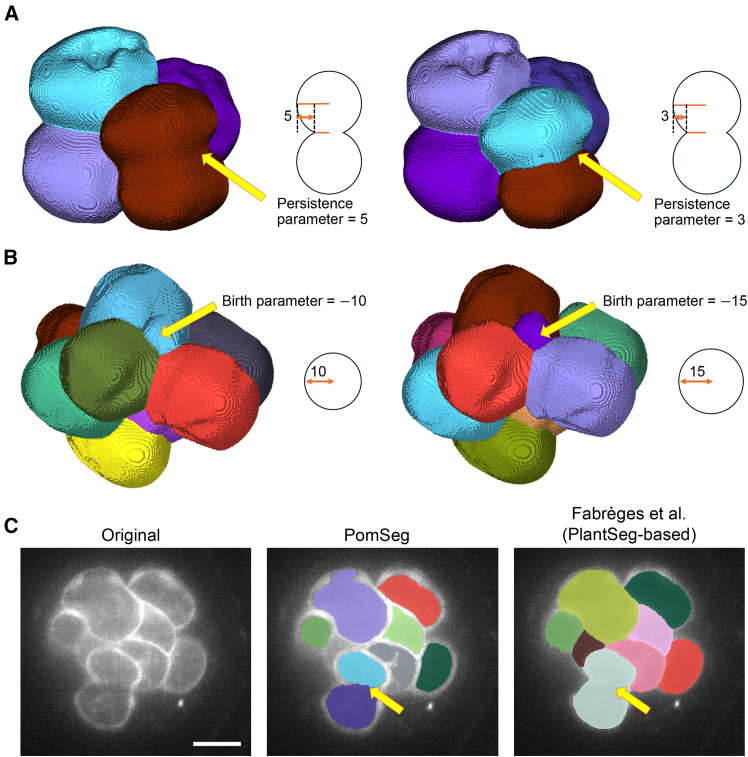
Flexibility of PomSeg enabled by its interpretable parameters (A) An example where we can adjust the PomSeg’s segmentation results by persistence parameter tuning (Table S1, 37th data). The left image shows that the structure highlighted by an arrow is processed as a single cell with persistence parameter 5. The right image shows that the same structure is processed as two contacting cells with persistence parameter 3. Labels after smoothing are shown. (B) An example where we can adjust the PomSeg’s segmentation results by birth parameter tuning (Table S2, 45th data). The left image shows that the small structure highlighted by an arrow is detected with birth parameter −10, while the right image shows that the same structure is merged with another larger structure with birth parameter −15. Labels after smoothing are shown. (C) An example where PomSeg and the ground truth segmentation give different segmentation results. The left image is the original image, the middle is the labels generated by PomSeg, and the right is the labels of the ground truth. The arrows highlight the difference between the two results. We can see that PomSeg captures 3D shape information (Table S1, 79th data). Scale bars, 25 μm. All the births in the figure mean the births of the points in the persistence diagrams.

PomSeg is especially helpful when we need good control over the detection of overlapping structures or objects with different sizes (Figures 6A and 6B). We can modify whether PomSeg merges or separates cells by increasing or decreasing the persistence parameter, as in Figure 6A. Similarly, the left side of Figure 6B shows that PomSeg detects a small structure. We can choose to ignore this structure by increasing the absolute value of the birth parameter, as in the right side of Figure 6B. These are the situations where the flexibility of PomSeg shines.

When the input images have ambiguous cell boundaries, such as the bottom cells in Figure 6C, PomSeg respects the 3D shape information and separately segments these cells (middle of Figure 6C), whereas the previous result,^35^ which has so far been used as the ground truth, integrates them (right of Figure 6C). This illustrates the efficacy of our topology-based method in separating cells with ambiguous boundaries.

### Extensibility of PomSeg demonstrated with multicellular stage embryo and different preprocessing

In this section, we show examples of the extensibility of PomSeg. Since PomSeg modules are all made with white-box processes, we can easily modify some parts of them or combine them with other tools.

First, we applied PomSeg to analyze multicellular stage embryo images (Figure 7A) in Ichikawa et al.^38^ with a slight modification. Here, we refer to the analysis results from Ichikawa et al.,^38^ obtained using PlantSeg with manual modifications, as our ground truth. As with the ground truth used in the previous sections, these results had been confirmed to be consistent with actual cells. In the 2D module, we enhanced the membrane parts by taking the contours of the masks using an OpenCV library in Python. In the 3D module, we conducted approximate segmentation using METIS,^45^ an equal-weight graph partitioning algorithm, and persistent homology, by first using METIS for rough partitioning and then applying persistent homology for finer shape-based separation. Although in multicellular stage images, the cell shapes are largely distorted and the individual segmentations are just approximations, we can use them to analyze cell numbers and volumes.

**Figure 7:**
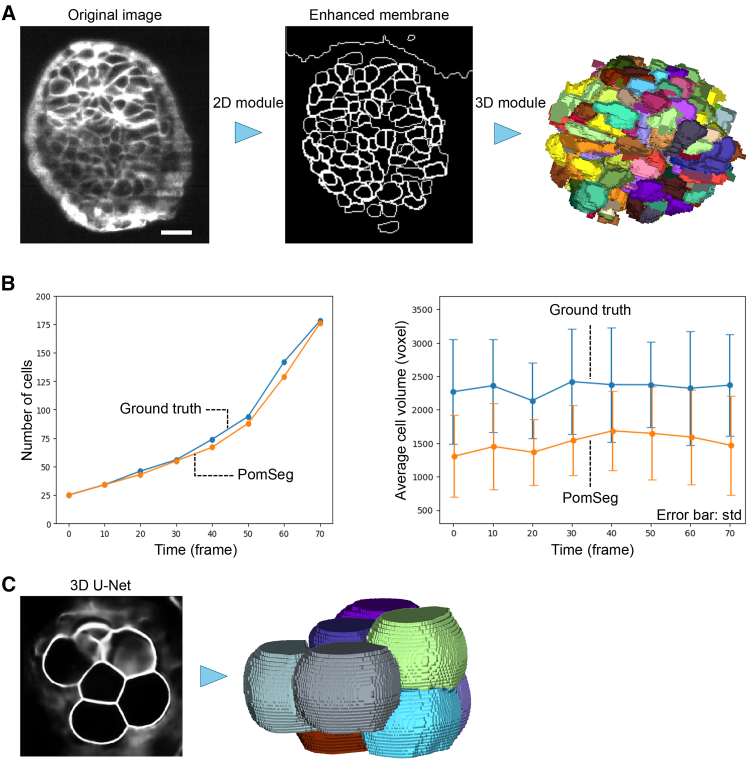
Extensibility of PomSeg demonstrated with different inputs (A) An illustration of the PomSeg pipeline for multicellular stage mouse embryo from Ichikawa et al.^38^ The left image is the original image, the middle is the result of the 2D module with contour drawn by an OpenCV library in Python, and the right is the labels generated by the 3D module. The resolution of the *xy*-plane is 0.83 μm/px, and the resolution of the *z* axis is 1.00 μm/px. Scale bars, 25 μm. (B) Comparisons of cell numbers (left) and volumes (right) between PomSeg result (orange) and the ground truth result (blue). The error bars of the right figure show the standard deviations. (C) A PomSeg segmentation result when given an input from a 3D U-Net preprocessing. Although the 3D U-Net boundary was qualitatively different from the Sato filter-generated boundary, PomSeg successfully handled this input and generated an appropriate segmentation. This shows that PomSeg can be effectively combined with machine learning-based tools.

PomSeg and the ground truth gave similar results for the analysis of cell numbers and average cell volumes for the 8 time points (Figure 7B). The relative error of cell count, which is the absolute difference in cell counts between the two methods divided by the count in the ground truth, was on average 0.04. Additionally, we tracked the segmented labels in the ground truth using the minimum weight maximum matching algorithm that we used in the 8-cell stage tracking. Some of the long-tracked tracking results showed a gradual increase and a drop pattern in the volume change, as visualized in Figure S4. This aligns with the regular cell volume change at each cell cycle reported in Ichikawa et al.^38^ This fact supports the validity of our tracking process in larger cell number stages.

Next, we tried different preprocessing for PomSeg. Sato filter is a simple and transparent tool without the complexity of a black-box nature. It handles boundary enhancement processing effectively, which motivated its use in the PomSeg preprocessing part. However, recent progress in machine learning-based tools, such as U-Net-based boundary prediction, may produce enhanced boundaries more automatically for pre-trained image data. Therefore, to help users select a better sub-tool depending on the situation, we tried applying a 3D U-Net-based boundary prediction instead of the Sato filter of a preprocessing step in PomSeg using the 53rd time frame in the 3D module section. We can see a qualitative difference between Sato filter-generated boundaries (Figure 2A) and 3D U-Net-generated boundaries (Figure 7C, left). Despite this difference, PomSeg was able to yield an accurate segmentation using the 3D U-Net input (Figure 7C, right). This result shows that it is possible to combine PomSeg with machine learning-based tools as its preprocessing step to aim for more automation while benefiting from PomSeg’s flexibility.

## Discussion

Using the framework of persistent homology, we developed PomSeg, a user-centered and flexible tool for segmenting cell membrane images in both 2D and 3D. The 2D module accepts a grayscale membrane image as input and processes it by calculating persistent homology based on pixel intensity values, resulting in a 2D segmentation output. The 3D module takes the slice-by-slice outputs from the 2D segmentation and stacks them to create a 3D binary object. It integrates distance transformation and 3D persistent homology to effectively capture the sizes of the objects and distinguish overlapping structures. The key advantage of PomSeg is its parameter interpretability. Specifically, the parameters correspond to pixel intensity differences in 2D and to cell size and overlap in 3D. Consequently, users can easily adjust the parameters based on the shape and size they want to recognize as cells in each input image, providing them with control for image analysis.

PomSeg 2D module was applied to images from different biological systems under varying conditions. It showed strong performance with relatively clean images. Even when dealing with images that had significant intensity variations, PomSeg enhanced the membrane regions effectively, although some errors were present. These results demonstrate the versatility of PomSeg. In the 3D analysis, we primarily applied PomSeg to images of 8-cell stage embryos and also to different developmental stages. Notably, we were able to analyze an entire set of 8-cell stage time series data using a single parameter set. PomSeg’s parameter tuning capability allows users to adapt to varying cell structures, especially overlapping cells, through processes of cell growth and division which extends its applicability to different cell stages. These results illustrate the robustness and flexibility of PomSeg. Furthermore, PomSeg demonstrated performance consistent with the ground truth in analyzing time series data for 8-cell stage embryos.

We also highlighted the robustness of PomSeg to variations in input resolution. This issue is particularly important, as researchers must carefully balance the trade-offs between high and low *z*-resolution when designing experiments. Higher *z*-resolution increases imaging time per frame and exposes samples to more light. Longer imaging time per frame may extend the interval between frames, while greater light exposure can shorten the overall imaging time span due to phototoxicity. Conversely, lowering the *z*-resolution can impair the accuracy of segmentation tools. PomSeg, which performs reliably at lower *z*-resolution, enables faster image acquisition and allows for longer time-lapse imaging.

It is important to note that parameter changes in PomSeg are reflected in the results without requiring the recomputation of persistent homology. In other words, once PomSeg has been applied to target images, users can skip recalculating the persistent homology step, a relatively computationally expensive process in PomSeg, when adjusting parameters for the same images. This enables faster adjustments, making PomSeg more user-friendly and efficient.

Another important point is that PomSeg and PlantSeg offer complementary approaches to segmentation, and this complementarity may enhance the flexibility of future workflows. PlantSeg generates an initial label using a watershed algorithm, typically resulting in oversegmentation, which is later refined by a graph-based merging process. In contrast, PomSeg’s 2D module produces a binary mask that separates cells from the background but does not resolve touching cells, effectively yielding undersegmentation. The 3D module then finalizes the segmentation by separating these touching cells based on topological features. While both methods follow a two-step design, coarse segmentation followed by refinement, they differ in how they handle initial segmentation errors: PlantSeg is suited to oversegmented inputs, whereas PomSeg excels with undersegmented ones. Thus, users encountering either issue can improve their results by selecting the appropriate tool, making the combination of PomSeg and PlantSeg a powerful and versatile strategy for segmentation tasks.

From a biological perspective, PomSeg expands the capabilities of persistent homology-based segmentation tools, which were previously limited to cell nuclei images, to include cell membrane images. This significantly broadens its applicability. Now, persistent homology-based segmentation tools cover two major bioimage modalities, thereby benefiting a wider range of biologists.

In summary, PomSeg stands out as a cutting-edge cell membrane segmentation tool grounded in persistent homology, offering significant advantages for users seeking precision and control in image analysis. Its flexibility in parameter tuning allows for user-centered customization, while its versatility ensures optimal performance across various image conditions. With a strong alignment to ground truth, PomSeg delivers reliable and consistent results, supported by its robustness to noise and different resolutions. Furthermore, the tool’s white box structure promotes extensibility, empowering users to build upon it and develop their own tailored solutions. By harnessing the full potential of PomSeg, users can elevate their data analysis capabilities, making it an indispensable asset in their research toolkit.

### Limitations of the study

A limitation of the PomSeg 3D module is its reliance solely on the shape information derived from the 3D binary image. If the boundary between two cells is perfectly spherical and lacks indentations, PomSeg may have difficulty separating them. This issue can arise when cell shapes are distorted due to them touching each other too much, or when lower *z-*resolution settings miss the cell contact slices, where the cell boundaries typically get narrow. In such cases, we will need to explore methods to incorporate additional information, such as data from adjacent time points, which is an area for future work. Another limitation is that extremely elongated cell shapes are difficult to distinguish from noise because cells are detected based on their short diameter (minimum radius). When facing these data-specific problems, PomSeg’s white box structure and extensibility will be helpful to make necessary modifications to suit specific needs.

## Resource availability

### Lead contact

Requests for further information and resources should be directed to and will be fulfilled by the lead contact, Haruhisa Oda (haruhisa-oda0722@g.ecc.u-tokyo.ac.jp).

### Materials availability

This study did not generate new unique reagents.

### Data and code availability

• Figure 2B fluorescence microscopy image of cells from “Image analysis with Python and Napari”: https://github.com/BiAPoL/HIP_Introduction_to_Napari_and_image_processing_with_Python_2022/tree/main or skimage.data.cells3d https://scikit-image.org/docs/stable/api/skimage.data.html
• Figure 2C plant cell image: https://osf.io/6x4gz
• Figure 2D epithelial cell image: https://github.com/tkphung/CellSegmentation/tree/main
• The sources of the embryo images are as follows: cell membrane images from mouse pre-implantation embryos were provided by Dr. Dimitri Fabrèges.^35^ Peri-implantation embryo images were provided by Dr. Takafumi Ichikawa.^38^
• PomSeg code is available at https://github.com/TopologicalBird/PomSeg, https://doi.org/10.5281/zenodo.18638747.
• PomSeg GUI standalone application can be downloaded from the link in the above repository.
• Any additional information required to reanalyze the data reported in this paper is available from the lead contact upon request.

## Acknowledgments

This work was supported in part by JST FOREST (JPMJFR222X to Y.I.) and JST CREST (JPMJCR24Q1 to Y.I.). H.O. would like to thank the University of Tokyo Medical Scientist Training Program and JST AIP challenge program for the financial support for this study. Some computation in this work was conducted using the UTokyo Azure (https://utelecon.adm.u-tokyo.ac.jp/en/research_computing/utokyo_azure/). The authors would also like to thank Prof. Shumpei Ishikawa and The University of Tokyo Preventive Medicine Laboratory for their support and suggestions.

## Author contributions

H.O. and Y.I. conceived the research idea and developed PomSeg through frequent discussions; H.O. developed the PomSeg code; H.O. drafted the manuscript; Y.I. improved the overall quality of the manuscript and suggested intuitive visualizations of mathematical concepts. All the authors reviewed the final version of the manuscript.

## Declaration of interests

At the time of submission, H.O. is affiliated with the University of Melbourne as well as the University of Tokyo. H.O. carried out this work solely as part of the affiliation with the University of Tokyo.

## Declaration of generative AI and AI-assisted technologies in the writing process

During the preparation of this work, the author(s) used Grammarly and ChatGPT in order to improve readability. After using this tool or service, the author(s) reviewed and edited the content as needed and take(s) full responsibility for the content of the publication.

## STAR★Methods

### Key resources table

**Table:** 

REAGENT or RESOURCE	SOURCE	IDENTIFIER
**Deposited data**		

fluorescence microscopy image of cells	GitHub	https://github.com/BiAPoL/HIP_Introduction_to_Napari_and_image_processing_with_Python_2022/tree/main
fluorescence microscopy image of cells	skimage.data.cells3d	https://scikit-image.org/docs/stable/api/skimage.data.html
plant cell image	OSF	https://osf.io/6x4gz
epithelial cell image	GitHub	https://github.com/tkphung/CellSegmentation/tree/main
Cell membrane images from mouse pre-implantation embryos	Fabrèges et al.^35^	https://doi.org/10.1126/science.adh1145
Cell membrane images from mouse peri-implantation embryos	Ichikawa et al.^38^	https://doi.org/10.1016/j.devcel.2021.12.023

**Software and algorithms**		

PomSeg	this paper	https://github.com/TopologicalBird/PomSeg; https://doi.org/10.5281/zenodo.18638747
PlantSeg	Wolny et al.^11^	https://doi.org/10.7554/eLife.57613
HomCloud	Obayashi et al.^46^	https://homcloud.dev/index.en.html
napari	napari contributors	https://doi.org/10.5281/zenodo.3555620

### Method details

PomSeg code was initially implemented in Jupyter Notebook with Python 3.10.9 and is also tested with Python 3.11.0. Persistent homology calculation was conducted using HomCloud^46^ available at https://homcloud.dev/index.en.html. For visualization of our time series image segmentation results, we used napari.^47^

#### PomSeg 2D module

The PomSeg 2D module processes 2D membrane images as input and produces 2D cell segmentation results. Initially, the input image undergoes preprocessing with a ridge filter (skimage.filters.sato https://scikit-image.org/docs/stable/api/skimage.filters.html#skimage.filters.sato) to enhance the pixel intensity of the membrane region. The resulting 2D grayscale image is then analyzed using 2D persistent homology, specifically applying sublevel set filtration.

From the resulting 0th persistence diagram, we select points in the persistence diagram with persistence values greater than a user-defined persistence parameter. This persistence reflects the intensity differences between the membrane and the interior of the cell. The persistence parameter sets the minimum intensity difference required for a structure to be detected.

For each selected point in the persistence diagram, we utilize the inverse analysis framework,^40^ which relates these points to structures within the input image. Since we are focusing on the 0th persistent homology, the structures identified through the inverse analysis are connected components. We can further refine our selection of relevant connected components using upper and lower bound values for their sizes. The resulting connected components are then used to generate a binary mask image for each input 2D membrane image. When we take contours of binary mask images, we use an OpenCV library in Python, cv2.findContours. For additional tips on mask construction and membrane enhancement, see Figure S5.

#### PomSeg 3D module

The PomSeg 3D module processes a 3D cell membrane image, which consists of a set of 2D membrane images at multiple *z*-slices. First, we apply the PomSeg 2D module to each slice individually to generate a set of 2D binary mask images. Next, we stack these 2D binary masks in the order of the original *z*-slices. If the resolution along the *z* axis differs from the *x*-*y* resolution, we approximate the resolution ratio using an integer. We then replicate the same slice for the number of times indicated by this integer when assembling the 2D binary masks. The output of this process is a 3D binary image where the *x*, *y*, and *z* axes have approximately the same resolutions, typically showcasing sphere-like overlapping cell structures.

To effectively segment these cells, we combine a distance transform process with a 3D persistent homology framework. The 3D distance transform is an image transformation method that takes a 3D binary image as input and produces a 3D grayscale image of the same dimensions. For each pixel in the binary image, we calculate its minimum distance to the boundary of the black (background) and white (foreground) regions. We assign these distances, with negative signs indicating foreground and positive signs indicating background, to form the output grayscale image. In our specific application to 3D binary cell images, we utilized the Manhattan distance defined as *d*((*x*, *y*, *z*), (*X*, *Y*, *Z*)) = |*x*-*X*|+|*y*-*Y*|+|*z*-*Z*|. Consequently, the central regions of the cells were assigned strongly negative values, while values near the cell surfaces approached zero. The outer regions received positive values.

Next, we apply a 3D sublevel set filtration persistent homology to the distance-transformed grayscale image. From the resulting 0th persistence diagram, we select points with persistence and the absolute value of birth larger than specified parameters, determined by the user. It is important to note that birth values are negative due to how our grayscale image was constructed using the distance transform. In this context, birth value reflects the first moment when the center of a cell enters the filtration, thus corresponding to the cell size. On the other hand, the persistence value indicates how long a structure remains before merging with another. Therefore, if a cell significantly overlaps with another, its persistence will be smaller, indicating the extent of overlap. For each selected point in the persistence diagram, we retrieve the birth position, which represents the first pixel that contributes to the point. This approach allows us to approximate the center of the cell. We then use these birth positions as markers for the watershed algorithm to finalize the 3D segmentation.

#### Smoothing

When smoothing the 3D segmentation generated above, we first shrink the segmentation by erosion process. Then, we use this as markers for the watershed algorithm, which is now applied to the original membrane image. Based on this refined segmentation, we calculate Gaussian filtered distance transform for each label. Finally, we get smoothed segmentation labels by assigning each voxel a label with the minimum Gaussian filtered distance.

#### Tracking of the segmented labels

Once we have segmented labels across all time points, we proceed with tracking as follows. First, we construct a bipartite graph from the segmented labels of adjacent time points. Since we are dealing with the 8-cell stage, our bipartite graph features 8 vertices in each part, resulting in a total of 16 vertices. Our edge set includes all permissible edges for a bipartite graph. We assign weights to each edge based on the distance between the barycenters of the connected cell labels. Next, we apply the minimum weight maximum matching algorithm to this weighted bipartite graph using the skimage.filters.sato in Python. The matched vertices are considered to represent the same cell across adjacent time points. By applying this process to all pairs of adjacent time points, we obtain the tracking results.

### Mathematical formulation of pomseg

#### Persistent homology overview

Given a grayscale image f:D→R, where *D* is the image region, its sublevel sets *X*_*r*_ = *f*^−1^(-*∞*,*r*] satisfy $X_{r_{1}}\subset X_{r_{2}}\subset D$ if *r*_1_<*r*_2_. Suppose that these sublevel sets change finitely many times at *r*_1_,*r*_2_, …,*r*_*n*_. We fix a base field K and consider the family of vector spaces formed by the *q*th homology groups ${\left\{H_{q}\left(X_{r_{i}}\right)\right\}}_{i=1,2,\ldots ,n}$. We have linear maps between these *q*th homology groups $\iota_{ij}:H_{q}\left(X_{r_{i}}\right)\to H_{q}\left(X_{r_{j}}\right)$, where *i* ≤ *j*, induced from inclusion maps. Persistence module is a finite family of vector spaces $W={\left\{W_{i}\right\}}_{i}$ indexed by natural numbers together with linear maps *f*_*ij*_:*W*_*i*_→*W*_*j*_ for *i* ≤ *j* such that *f*_*ik*_ = *f*_*jk*_∘*f*_*ij*_ for *i* ≤ *j* ≤ *k* and *f*_*ii*_ = id for all *i*. Interval module $K_{I}$ for an interval *I* is a special case of persistence module where ${\left(K_{I}\right)}_{i}=K$ for *i*∈*I* and ${\left(K_{I}\right)}_{i}=0$ otherwise. Linear maps are identities inside *I* and zeros otherwise. Interval decomposition theorem states that persistence module can be uniquely decomposed into interval modules. Since the above family of *q*th homology groups with the linear maps induced from inclusions satisfies the criterion for persistence module, it can be decomposed into interval modules. We visualize this decomposition by plotting each interval’s left and right ends in the *x*-*y* coordinate. This visualized 2D plot is called the persistence diagram.

#### Input data

We denote by [*k*] a set {0,1, …,*k*-1}. Our main application target, the sequence of cell images ${\left\{I_{z}\right\}}_{z\in \left[L\right]}$, has a 2D slice *I*_*z*_ of size *M*×*N* for each *z* slice. We can think of *I*_*z*_ as a function $I_{z}:D\to R_{\geq 0}$, where *D*≔[*M*]×[*N*].

#### PomSeg 2D module

##### Preprocessing

We preprocess each 2D slice *I*_*z*_ using a ridge filter (skimage.filters.sato) to get a processed 2D slice $J_{z}:D\to R_{\geq 0}$. This preprocessing enhances the membrane intensities. We then slightly modify *J*_*z*_ as

$$J_{z}^{\prime }\left(x,y\right)=\left\{ \begin{array}{c} J_{z}\left(x,y\right)+1\;\text{if}\;\left(x,y\right)\neq \left(x_{0},y_{0}\right),\\ 0\;\text{if}\;\left(x,y\right)=\left(x_{0},y_{0}\right). \end{array}\right.$$

Here, (*x*_0_,*y*_0_) is a point in the background. This modification ensures that the region that includes (*x*_0_,*y*_0_), namely the background, goes to the essential class in the process described in the next section. In this paper, the upper left corner of the image does not have cells, so we use it as a point in the background.

##### 2D persistent homology

We apply sublevel filtration persistent homology to $J_{z}^{\prime }$. From the resulting 0th persistence diagram $PD=\left\{\left(b_{i},d_{i}\right)|\;i\in \Lambda \right\}$ (*Λ*: index set), we use the persistence parameter, *t*_p_, to select the points in the persistence diagram ${PD}_{t_{p}}=\left\{\left(b_{i},d_{i}\right)\in PD|\;t_{p}<d_{i}-b_{i},d_{i}<\infty \right\}$. For each point in the persistence diagram (birth-death pair) $\left(b,d\right)\in {PD}_{t_{p}}$, we calculate the optimal volume *V*_(*b*,*d*)_⊂*D*, which is the maximal connected component corresponding to the pair. We set the volume size threshold values, *t*_v_ and *t*_V_, to select the set of valid volumes $V=\left\{V_{\left(b,d\right)}|\;t_{v}<\#V_{\left(b,d\right)}<t_{V}\right\}$ to generate a mask image. Based on this, we define the binary mask image *M*:*D*→{0,1} by *M*(*x*,*y*) = 1if there exists some V∈V such that (*x*,*y*)∈*V* and *M*(*x*,*y*) = 0 otherwise. Before moving to the 3D module, we can apply noise reduction as shown in Figure S3 when we have noise inside the cells.

#### PomSeg 3D module

##### 3D binary image construction

Based on the outputs of the 2D analysis part, we construct a 3D binary image as follows:

For the slice index *z*∈[*L*], let *M*_*z*_:*D*→{0,1} be a *z*th 2D binary mask obtained from the PomSeg 2D module. When the interval of *z*-slices is larger than the pixel sizes of the *xy* plane *D*, (namely, *z*-resolution is lower than *xy*-resolution), we adjust the sequence of *M*_*z*_ following the ratio of them. Let *r* be the closest integer of the ratio (interval size of *z*-slices divided by pixel sizes). Then, we define the adjusted binary 3D image *B*:*D*×[*rL*]→{0,1} by *B*(*x*, *y*, *z*)≔*M*_⌊*z*/*r*⌋_(*x*, *y*) where ⌊*n*⌋ is the greatest integer less than or equal to *n*. This procedure makes the adjusted 3D image close to isotropic, which allows the next distance transform process to be less affected by the resolution difference. In this study, we chose *r* = 3.

##### Distance transform

Next, we apply a distance transform to this 3D binary image. The distance transform process can be described as follows: The input binary image has voxels with colors white or black. We denote by *V*_B_ and *V*_W_ the sets of voxels with colors black and white, respectively. For each voxel (*x*_1_, *y*_1_, *z*_1_)∈*V*_B_ or (*x*_2_, *y*_2_, *z*_2_)∈*V*_W_, we assign the distance min⁡{*d*((*x*_1_, *y*_1_, *z*_1_),(*x*, *y*, *z*))| (*x*, *y*, *z*)∈*V*_W_} or -min⁡{*d*((*x*_2_, *y*_2_, *z*_2_), (*x*, *y*, *z*))| (*x*,*y*,*z*)∈*V*_B_}, where *d* is the distance we use. We mainly use Manhattan distance *d*((*x*, *y*, *z*),(*X*, *Y*, *Z*)) = |*x*-*X*|+|*y*-*Y*|+|*z*-*Z*|. Now, we define the distance transformed image $T_{B}:D\times \left[rL\right]\to R$ by *T*_*B*_(*x*, *y*, *z*) = min⁡{*d*((*x*,*y*,*z*),(*x*′,*y*′,*z*′))| (*x*′, *y*′, *z*′)∈*V*_W_} if (*x*, *y*, *z*)∈*V*_B_ and *T*_*B*_(*x*, *y*, *z*) = -min⁡{*d*((*x*, *y*, *z*), (*x*′, *y*′, *z*′))| (*x*′, *y*′, *z*′)∈*V*_B_} if (*x*, *y*, *z*)∈*V*_W_. This distance transform process provides the following persistent homology process with size scale information.

##### 3D persistent homology and finalizing segmentation

We apply sublevel set filtration persistent homology to *T*_*B*_. From the resulting 0th persistence diagram $PD=\left\{\left(b_{i},\;d_{i}\right)|\;i\in \Lambda \right\}$ (*Λ*: index set), we use persistence and birth parameters, *T*_p_,*T*_b_, to select points in the persistence diagram ${PD}_{T_{p},T_{b}}=\left\{\left(b_{i},\;d_{i}\right)\in PD|\;T_{p}<d_{i}-b_{i},b_{i}<T_{b}\right\}$. For each birth-death pair in ${PD}_{T_{p},T_{b}}$, we retrieve the birth position, which represents the first structure that gave birth to the pair. We apply watershed method (skimage.segmentation.watershed) to *T*_*B*_ with markers being the birth positions and the mask being *B*. This watershed process starts from the markers we prepared and floods basins until basins for different markers meet. Now, we get the segmented labels.

##### Tracking of the segmented labels

Given the segmented labels for the whole time points, we construct a bipartite graph from the two segmented labels in adjacent time points. At the 8-cell stage, our bipartite graph has 8 vertices in each part (16 vertices in total, denoted by {*v*_0_,…, *v*_7_} and {*w*_0_,…, *w*_7_}) corresponding to the 8 cells in each time point. Our edge set is {(*v*_*i*_, *w*_*j*_)| *i*,*j*∈{0, …,7}}, where (*v*_*i*_, *w*_*j*_) stands for the edge connecting the vertices *v*_*i*_ and *w*_*j*_. We give weights to the edges (*v*_*i*_, *w*_*j*_) by the distances between the barycenters of the *i*th cell in the first time point and the *j*th cell in the second time point. We apply the minimum weight maximum matching algorithm to this weighted bipartite graph using networkx.min_weight_matching. We regard the matched vertices represent the same cell in the adjacent time points. This gives us the tracking result.

### Quantification and statistical analysis

To evaluate the time variations of persistences *P*_2_-*P*_10_ in Figure 3C, we calculated the pairwise distance between *P*_*i*_ and *P*_*j*_ using the *L*^1^norm, defined as $d\left(P_{i},\;P_{j}\right)=\sum_{t}|P_{i}\left(t\right)-P_{j}\left(t\right)|$. We then analyzed the resulting distance matrix by applying multidimensional scaling (MDS) to visualize the distribution of the persistences *P*_*i*_ (Figure S2B).

For segmented cell volume, we assessed it by counting the total number of voxels associated with the same segmentation label (Figure 4A).

To evaluate volume variation, we calculated the coefficient of variation, which is defined as the standard deviation divided by the average volume. We performed this calculation for the 8 cells and then averaged the 8 coefficients of variation.

The 3D U-Net used in Figure 7C was taken from PlantSeg.^11^

## Supplemental information

Document S1. Figures S1–S5 and Tables S1 and S2

Document S2. Article plus supplemental information
