# Supplementary material for: Persistent homology-based segmentation tool for membrane images
Source: Cell Rep Methods. 2026 Mar 26;6(4):101366. doi: 10.1016/j.crmeth.2026.101366 (PMC13107053; doi:10.1016/j.crmeth.2026.101366)
Supplement: Document S1. Figures S1–S5 and Tables S1 and S2 [file mmc1.pdf]

Cell Reports Methods, Volume *6*

Supplemental information

Persistent homology-based segmentation tool for membrane images

Haruhisa Oda and Yusuke Imoto

Oda-Imoto, Figure S1. Internal noise in 2D module

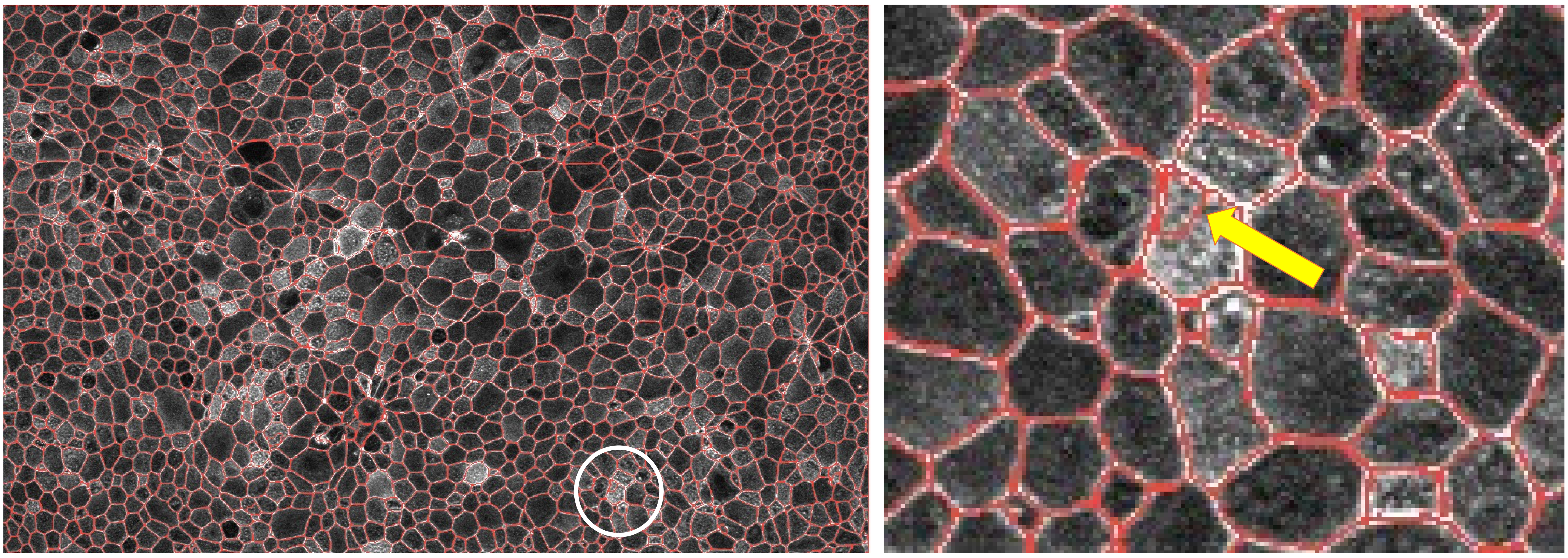

Oda-Imoto, Figure S2. Distributions of persistences

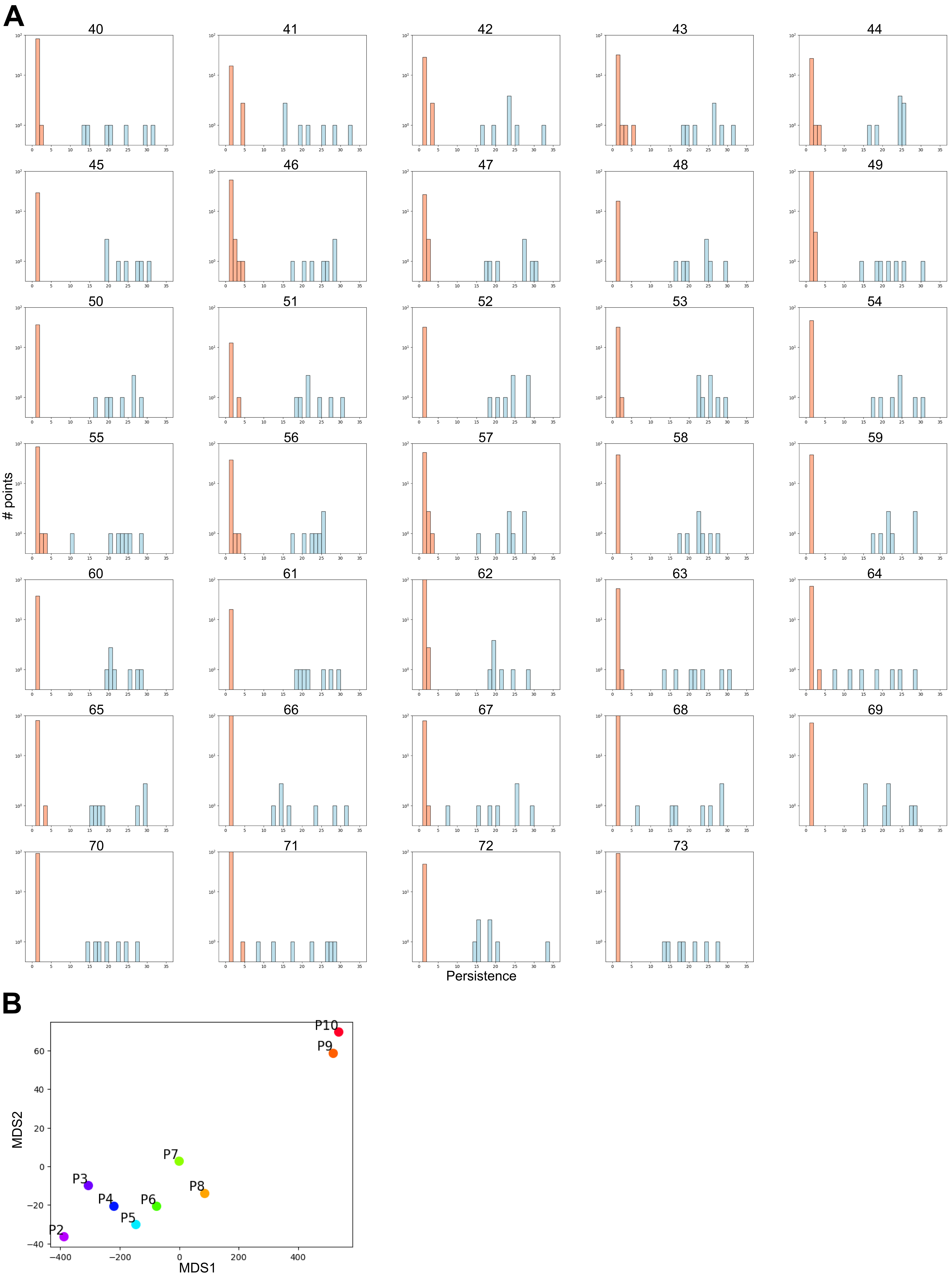

Oda-Imoto, Figure S3. Typical noise and their reduction

A

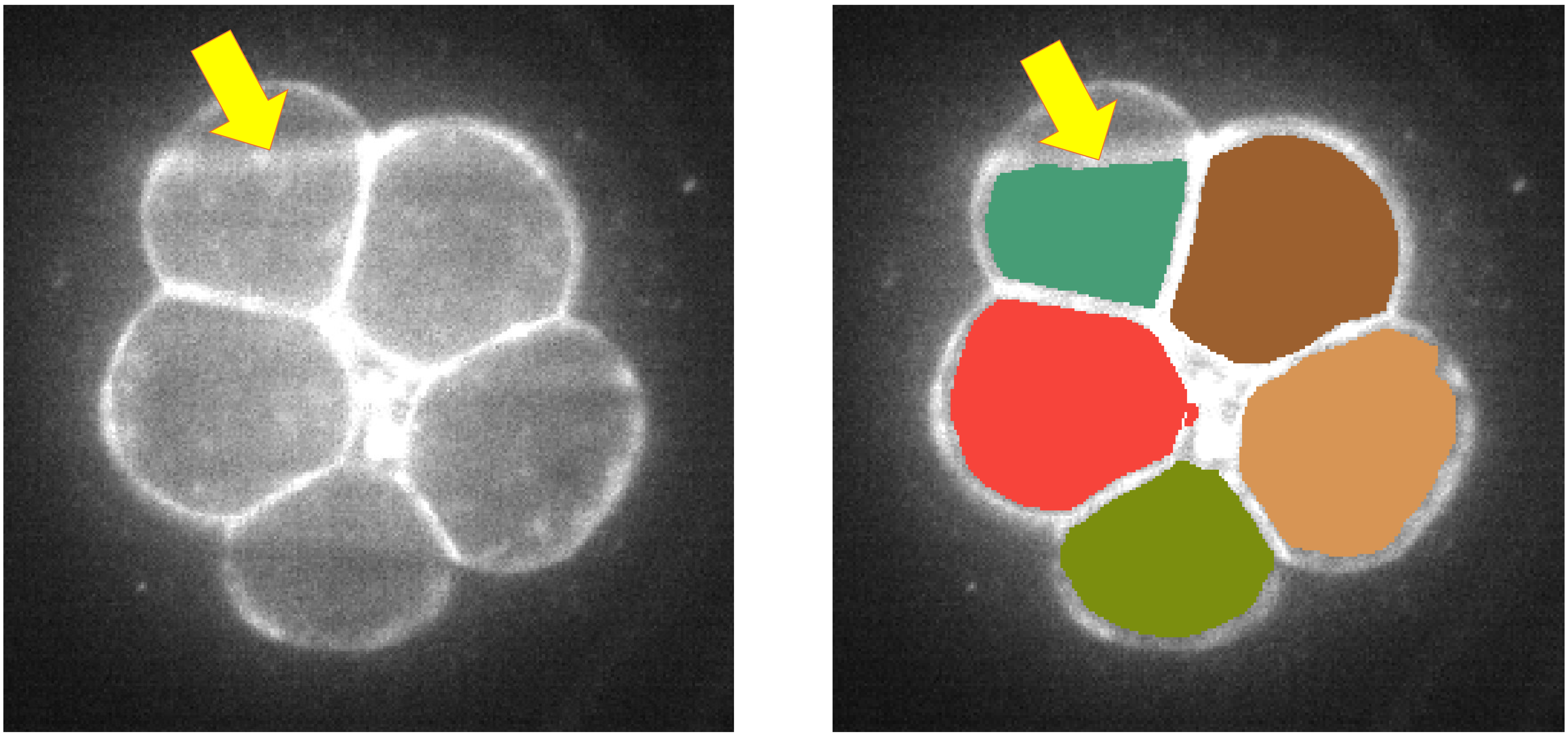

B

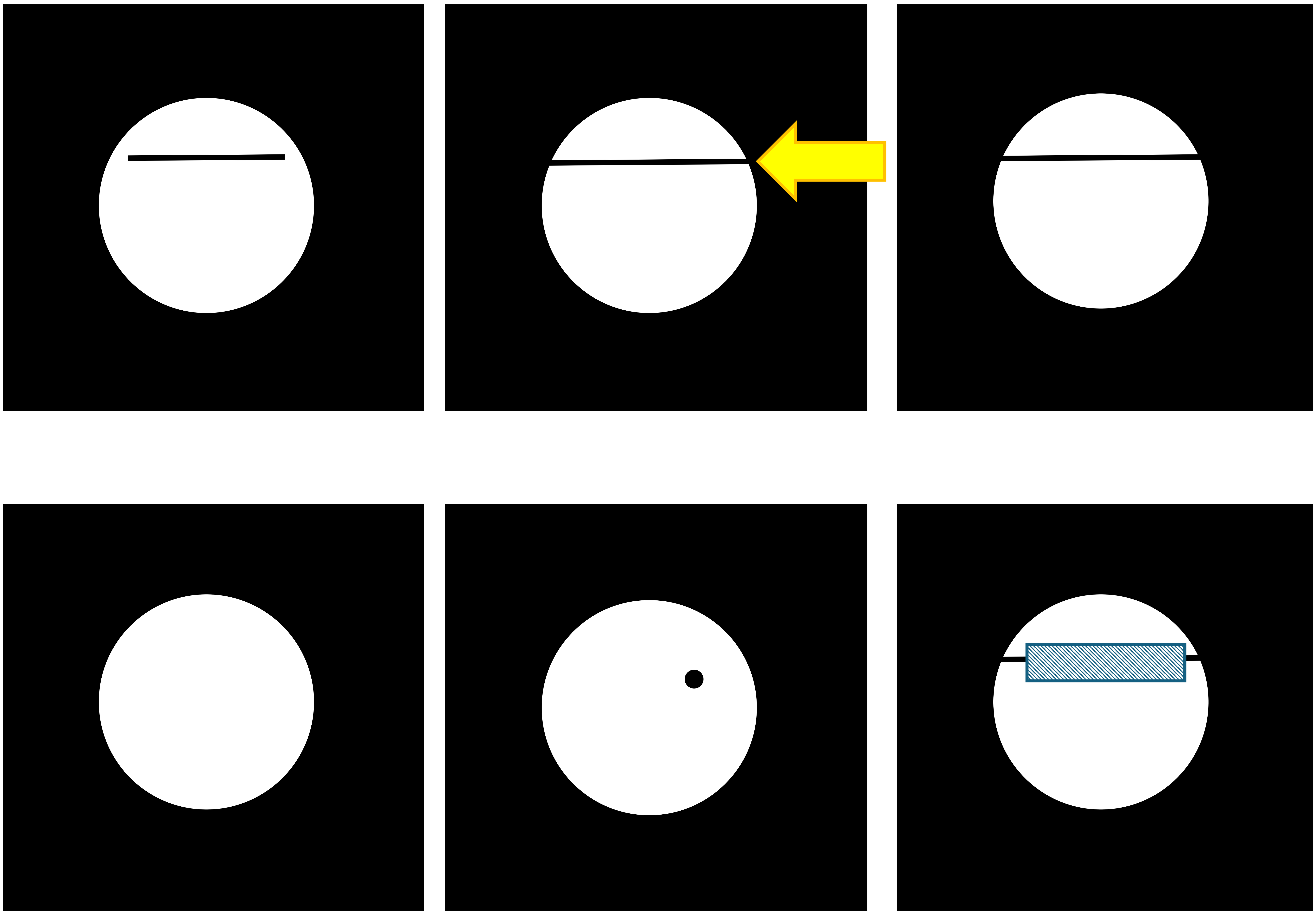

Oda-Imoto, Figure S4. Tracking of the segmented labels

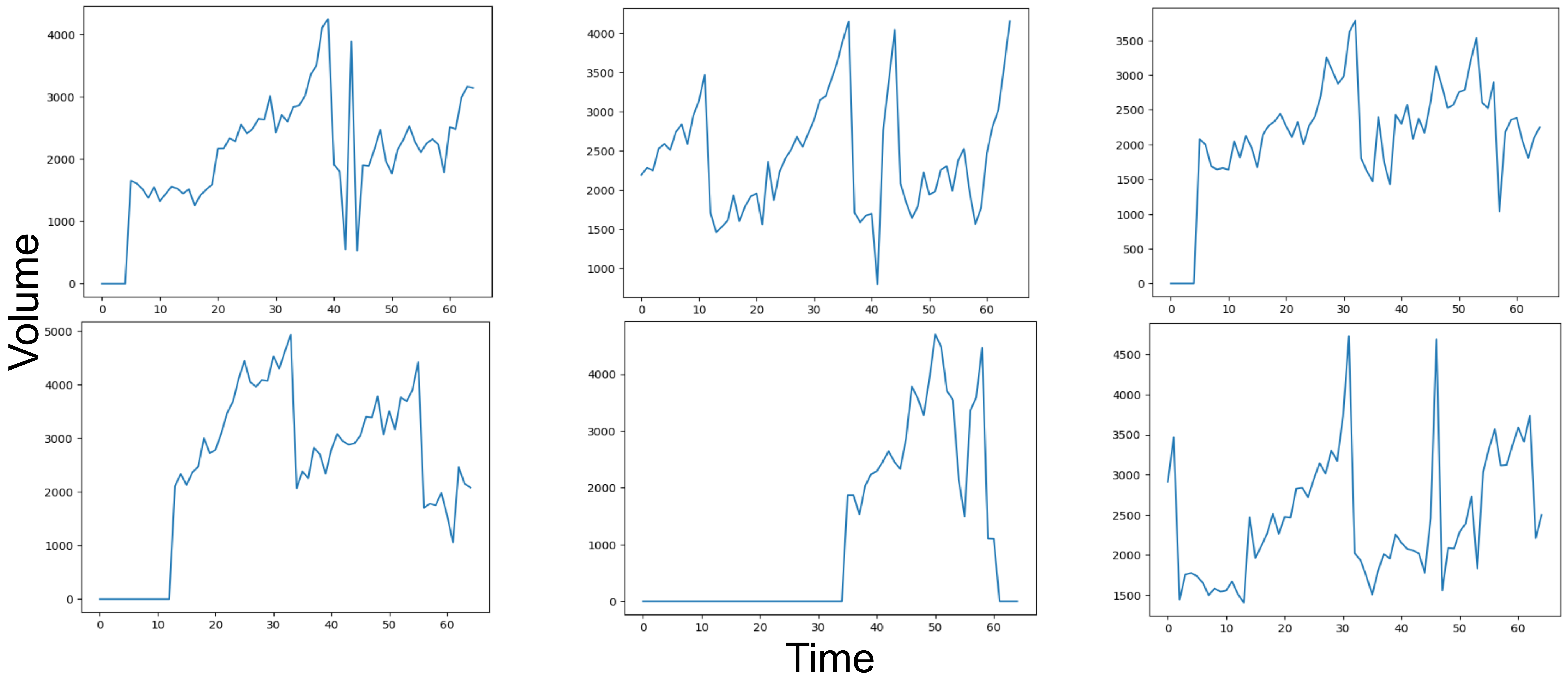

Oda-Imoto, Figure S5. Mask construction and membrane enhancement

A

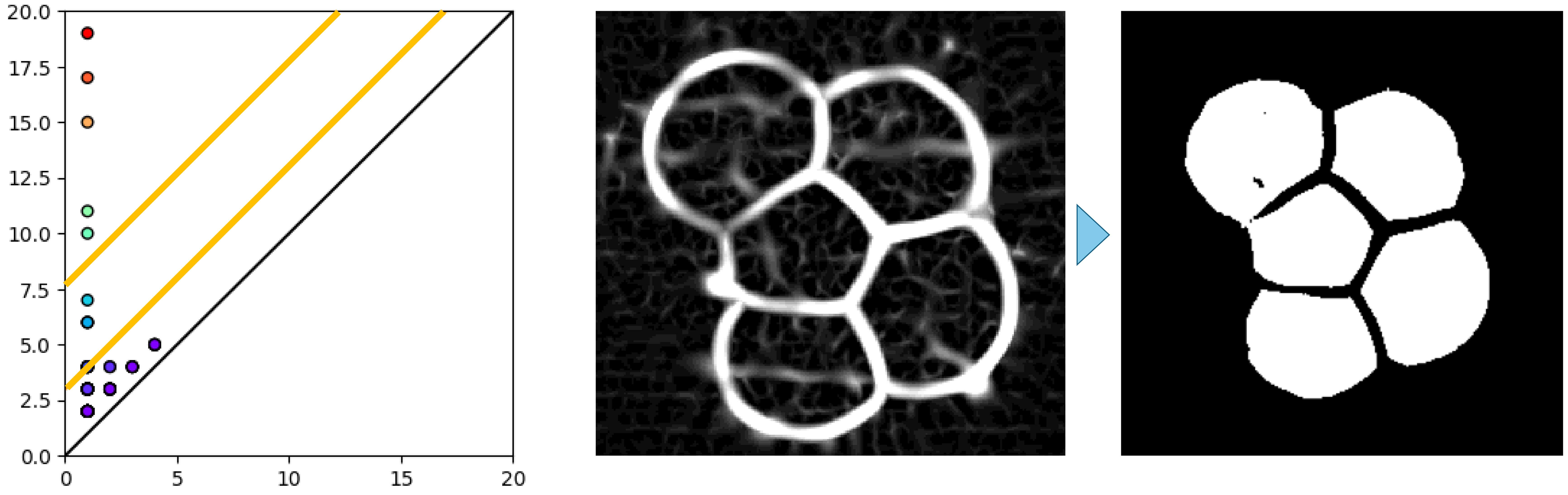

B

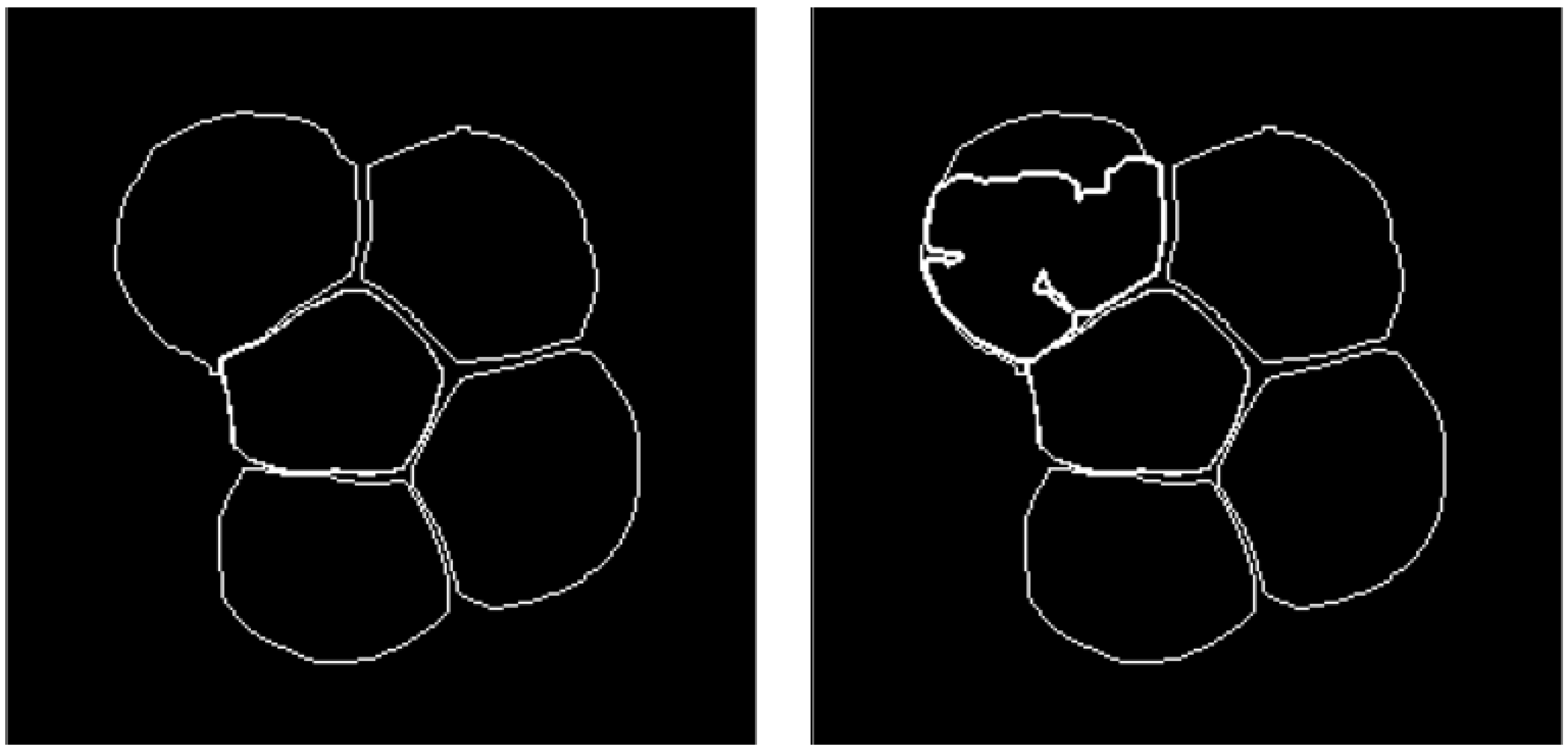

Figure S1. Internal noise in 2D module, related to Figure 2.

An internal noise detected in Figure 2D.

Figure S2. Distributions of persistences, related to Figure 3.

A) Histograms showing the distribution of persistence. The number written on each histogram is the time point index.

B) An MDS scatter plot of P_2– P_{10} with L^1 distance. The colors correspond to those in Figure 3C.

Figure S3. Typical noise and their reduction, related to Figure 6.

A) Typical noise in embryo images is the strong line shaped noise. We sometimes get this noise reflected in our mask. This noise can

- affect 3D segmentation process (e.g. the noise in the center part of the cell might affect persistence)
- affect the visual quality of resulting segmentations

B) In addition to simple morphological operations such as dilations, various noise reduction processes are available

1. External contour detection (slice by slice) focusing on the external contour.
2. External contour detection from different direction (slice by slice). Seen from different directions, the line looks like a point. Now we can erase this as in 1.
3. Manual noise reduction (we did not use it in the manuscript, but since the noise is usually line shaped in this data, we can erase it by drawing rectangular body. GUI for this can be made using napari.)

Figure S4. Tracking of the segmented labels, related to Figure 7.

Tracking of the segmented labels in the previous study using the minimum weight maximum matching algorithm that we used in the 8-cell stage tracking. Some of the long-tracked tracking results show a regular cell volume change corresponding to cell cycle.

Figure S5. Mask construction and membrane enhancement, related to STAR Methods.

A) In 2D mask construction, two persistence parameters (large/small) do not change the result; smaller structures inside larger structures do not appear in the final mask. This reduces the parameter tuning effort for 2D module.

B) When enhancing membranes, we can modify our process so that the above parameter change will change the result; internal line structures detected or not. Left: larger persistence parameter. Right: smaller persistence parameter.

These two processes are only a slight modification in the code. However, knowing this difference might help users interact with the code more effectively.

Table S1. Parameters used for the first additional data and the resulting counts (related to Figure 6)

Time point	35	37	39	40–73	74	75	76	77	78	79
Ground truth cell number	4	4	7	8	8	10	10	10	10	11
**PomSeg parameters**										
Preprocessing	Sato (2,5,1)	Sato (2,5,1)	Sato (2,5,1)	Sato (2,5,1)	Sato (2,5,1)	Sato (2,5,1)	Sato (2,5,1)	Sato (2,5,1)	Sato (2,5,1)	Sato (2,5,1)
2D persistence parameter	3	3	3	3	3	3	3	3	3	3
Volume threshold	2000	2000	2000	2000	2000	2000	1000	2000	2000	1000
Noise reduction External contour	+	+	+	+	+	+	+	+	+	+
Noise reduction Dilation with kernel1	1	1	1	1	1	1	1	1	1	1
Birth parameter (3D)	−10	−10	−10	−10	−10	−10	−10	−10	−10	−10
Persistence parameter (3D)	5	3/5	5	5	5	4	4	5	2	4
Cell number by **PomSeg**	4	5/4	7	8	8	10	10	10	10	12

Table S2. Parameters used for the second additional data and the resulting counts (related to Figure 6)

Time point	25	28	30	35	40	45	50	55	60	65	70	75
Ground truth cell number	4	5	6	7	8	8	8	8	8	9	10	11
**PomSeg parameters**												
Preprocessing	Sato (3,7,1)	Sato (3,7,1)	Sato (3,7,1)	Sato (3,7,1)	Sato (3,7,1)	Sato (3,7,1)	Sato (3,7,1)	Sato (3,7,1)	Sato (3,7,1)	Sato (3,7,1)	Sato (3,7,1)	Sato (3,7,1)
2D persistence parameter	2	2	2	2	2	2	2	2	2	2	3	3
Volume threshold	2000	1000	2000	2000	2000	2000	2000	2000	2000	1000	1000	1000
Noise reduction External contour	+	+	+	+	+	+	+	+	+	+	+	+
Noise reduction Dilation with kernel1	1	1	1	1	1	1	1	1	1	1	2	1
Noise reduction Dilation with kernel2	0	0	0	0	0	0	0	0	0	0	2	0
Noise reduction External contour from a different direction	−	+	+	+	+	−	+	+	+	−	−	−
Birth parameter (3D)	−10	−10	−10	−10	−10	−15	−20	−10	−10	−18	−10	−10
Persistence parameter (3D)	5	5	5	5	7	4	3	1	1	2	2	3
Cell number by **PomSeg**	4	5	6	7	8	8	8	8	8	9	10	11

kernel1=[[1,1,1],[1,1,1],[1,1,1]]
kernel2=[[0,1,0],[1,1,1],[0,1,0]]
